# Neuroprotective Effect and Possible Mechanisms of Berberine in Diabetes-Related Cognitive Impairment: A Systematic Review and Meta-Analysis of Animal Studies

**DOI:** 10.3389/fphar.2022.917375

**Published:** 2022-06-06

**Authors:** Yanwei Hao, Jiaxin Li, Shengnan Yue, Shaofeng Wang, Shuangyuan Hu, Bin Li

**Affiliations:** ^1^ School of Clinical Medicine, Chengdu University of Traditional Chinese Medicine, Chengdu, China; ^2^ Hospital of Chengdu University of Traditional Chinese Medicine, Chengdu, China

**Keywords:** diabetes, cognitive impairment, berberine, animal models, systematic review, meta-analysis

## Abstract

Berberine, the main bioactive component of *Coptis chinensis* Franch., is widely used in the treatment of diabetes. Previous studies have reported that berberine supplementation may play a multitarget therapeutic role in diabetes-related cognitive impairment (DCI). This systematic review and meta-analysis evaluated the effect and possible mechanisms of berberine in animal models of DCI. Relevant studies were searched through PubMed, Web of Science, Embase, and three Chinese databases (CNKI, Wanfang, and VIP) until March 2022. Twenty studies involving 442 animals were included, and SYRCLE’s risk of bias tool was used to assess methodological quality. The statistical analysis was performed using STATA 15.0 to calculate the weighted standard mean difference (SMD) with a 95% confidence interval (CI). The fasting blood glucose (FBG) and Morris water maze test (MWM) were the main outcomes to be analyzed. The overall results showed that berberine could significantly improve FBG, escape latency, the times of crossing the platform, the time spent in the target quadrant, serum insulin, 2hBG of oral glucose tolerance test (OGTT), amyloid β (Aβ), acetylcholinesterase (AChE), oxidative stress, and inflammation levels. The present meta-analysis demonstrated that berberine could not only lower blood glucose levels but also improve learning and memory in DCI animal models, which might involve regulating glucose and lipid metabolism, improving insulin resistance, anti-oxidation, anti-neuroinflammation, inhibiting endoplasmic reticulum (ER) stress; and improving the cholinergic system. However, additional attention should be paid to these outcomes due to the significant heterogeneity.

## 1 Introduction

Diabetes mellitus (DM) has become one of the worldwide public health challenges in the twenty-first century (Giannini et al., 2014). According to the International Diabetes Federation Diabetes Atlas, in 2021, more than half a billion people worldwide suffer from diabetes, and the number is estimated to reach 782 million by 2045 (Sun et al., 2022). Diabetes-related cognitive impairment (DCI), a cognitive impairment caused by diabetes, is mainly characterized by declining learning and memory function (Mijnhout et al., 2006; Biessels and Reijmer, 2014). This is one of the chronic complications of diabetes. Studies showed that chronic diabetes could accelerate and promote the onset of neurodegeneration, and 29% of patients with DM will eventually develop severe cognitive decline and neurodegeneration (de Matos et al., 2018). A meta-analysis of 144 studies showed that DM increased the risk of mild cognitive impairment and dementia in the elderly by 1.25–1.91-fold (Xue et al., 2019). Multiple lines of evidence indicate that cognitive impairment is directly associated with DM and is a serious complication of diabetes (Cukierman et al., 2005; Biessels and Despa, 2018). The pathogenesis of DCI has been widely reported in recent years, including disorders of glucose and lipid metabolism, insulin resistance, oxidative stress, inflammatory response, endoplasmic reticulum (ER) stress, and blood–brain barrier damage (Karvani et al., 2019; Zhang et al., 2020). Unfortunately, there have been no specific drugs or therapies to delay the progression of cognitive impairment in diabetes so far. New therapies and drugs are urgently needed to prevent and treat diabetes-induced cognitive impairment.

**FIGURE 1:**
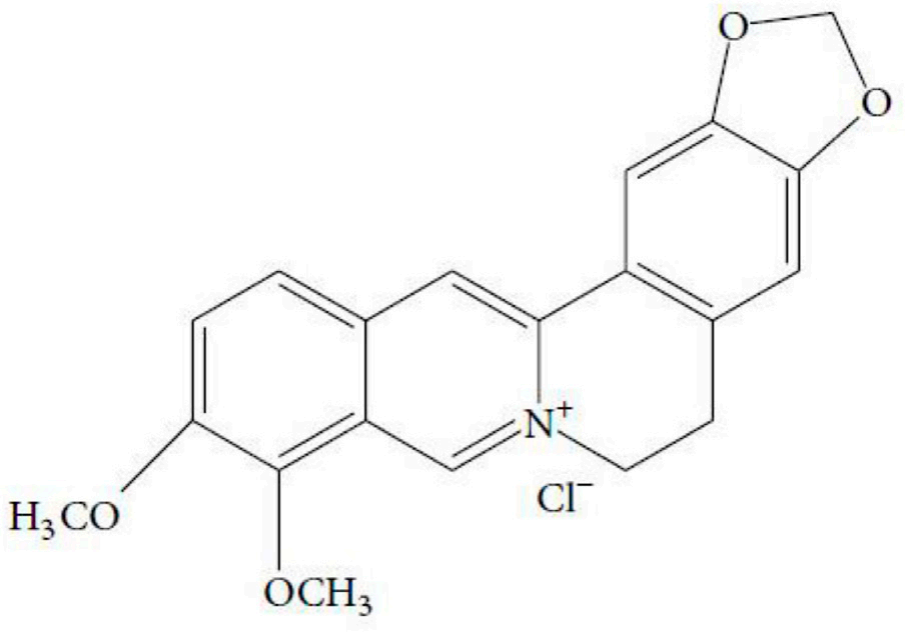
The chemical structure of berberine.

*Coptis chinensis* Franch. (Ranunculaceae), a recognized traditional herb in China, has been widely used in the treatment of type 2 diabetes mellitus (T2DM), hyperlipidemia, and gastrointestinal tract infection (Pang et al., 2015; Liu R. Y. et al., 2018). Berberine (C_20_H_18_NO_4_^+^, Figure 1) is the main bioactive component of *Coptis chinensis* Franch. (Zhao et al., 2021). In the modern pharmacological studies, due to its potential therapeutic applications, berberine has been extensively studied and has been shown to have multiple bioactivities, such as regulating glucose and lipid metabolism (Yin et al., 2008), anti-oxidation and anti-inflammation (Ma et al., 2018), and anti-cancer and neuroprotective effects (Song et al., 2020). As an isoquinoline alkaloid extracted from *Coptis chinensis*, berberine can effectively improve the cognitive function of DCI and Alzheimer’s disease (AD) (Shinjyo et al., 2020; Akbar et al., 2021). However, there is no meta-analysis based on preclinical studies to synthesize the evidence relating to berberine for the treatment of cognitive dysfunction in diabetes. This study aimed to conduct a systematic review and meta-analysis to analyze the neuroprotective effect and mechanisms of berberine on DCI by pooling relevant animal studies.

## 2 Methods

The Preferred Reporting Items for Systematic Reviews and Meta-Analysis (PRISMA) was followed to design and conduct the current systematic review and meta-analysis (Page et al., 2021).

### 2.1 Search Strategy

The databases of PubMed, Web of Science, Embase, China National Knowledge Infrastructure (CNKI), Wanfang Data Knowledge Service Platform, and VIP Periodical Service Platform were searched from database inception until March 2022. The search had no language restrictions. The combination of MeSH and free-text terms was used to identify the disease, intervention drug, and animals: [(“Diabetes Mellitus” OR “Diabetes, cognitive impairment” OR “Diabetes, cognitive dysfunction”) AND (“Berberine” OR “Umbellatine”) AND (“Animals” OR “Animal model” OR “Rat” OR “Mice”)].

### 2.2 Inclusion and Exclusion Criteria

The pre-determined inclusion criteria were as follows: 1) model: DM was selected as the research model. The success criterion of the models was blood glucose (BG) ≥ 11.1 mmol/L. 2) Intervention: the treatment group only received any dose of berberine. The control group was administered with an equal dose volume of nonfunctional sterile liquids or no treatment. 3) Outcome: the primary outcomes were fasting blood glucose (FBG) and behavioral tests. Behavioral tests were used to assess animal learning and memory function, such as the Morris water maze test (MWM), Y-maze test, passive avoidance test, and conditioned fear test. The secondary outcomes were serum insulin, hemoglobin A1c (HbA1c), 2 h blood glucose (2hBG) of oral glucose tolerance test (OGTT), 2hBG of insulin tolerance test (ITT), and the related mechanisms of berberine against DCI. The exclusion criteria were as follows: 1) experimental studies unrelated to impairment of learning, memory, and cognitive function; 2) no control group or treatment group without berberine or combined with other intervention; 3) non-*in vivo* studies; 4) no full texts, and 5) duplicate data or publications.

### 2.3 Data Extraction

All retrieved literature was imported into EndNote X9 and eliminated duplicates. Two researchers (L.J.X and W.S.F) independently performed literature collection based on the inclusion and exclusion criteria. First, the title and abstract were screened to exclude irrelevant articles. Second, the remaining articles were reviewed by obtaining the full text. The following details were extracted from selected studies: 1) name of first author and year of publication; 2) characteristics of experimental animals, including animal species, sex, sample size, age, and weight; 3) modeling methods and the criteria for modeling successfully; 4) drug intervention, including administration method, drug dose, and duration of treatment; and 5) outcome indicators and intergroup differences, including blood glucose index, cognitive assessment, and mechanism of action. We created a database and manually extracted data from the included literature. If the results were shown only by graphs, we tried to contact the author for more information. The graph data were quantified by WebPlotDigitizer 4.5 software (https://automeris.io/WebPlotDigitizer) if there was no response. For the results obtained at multiple time points, only the data of the last time point before the animals were sacrificed were extracted. If the drug involved several doses in the treatment group, we only extracted data for the highest dose.

### 2.4 Risk of Bias Assessment

We used the SYRCLE risk of bias tool based on the Cochrane risk of bias tool to assess the methodological quality of the included studies (Hooijmans et al., 2014). The tool mainly covers selection bias, performance bias, detection bias, reporting bias, and other biases. The maximum score is 10 for each study. Any divergences in the process of quality assessment were finally resolved by consultation with the correspondence author.

### 2.5 Data Synthesis and Analysis

The statistical analysis was performed using STATA software version 15.0. The outcome of each indicator was considered a continuous variable, and the combined overall effect sizes of outcome were expressed using the standard mean difference (SMD) and 95% confidence interval (CI). It is considered that there is a significant difference between the treatment group and the control group when *p* < 0.05. *I*-square (*I* ^2^) was used to evaluate statistical heterogeneity. The fixed effects model was used to combine the effect size when *I* ^2^ ≤ 50%, and the random effects model was used to combine the effect size when *I* ^2^ > 50%. In order to recognize the source of heterogeneity among included studies, subgroup analysis based on year of publication, animal species, the dose of STZ (≤ 40 and > 40 mg/kg), dose of berberine (≤ 100 and > 100 mg/kg), and duration of treatment (≤ 8 and > 8 weeks) were conducted. Then, sensitivity analysis was performed to evaluate the stability of the overall results. Potential publication bias was assessed using Egger’s test if the number of included datasets was 10 or higher. Moreover, the trim and fill method was performed in the presence of publication bias.

## 3 Results

### 3.1 Study Inclusion

**FIGURE 2:**
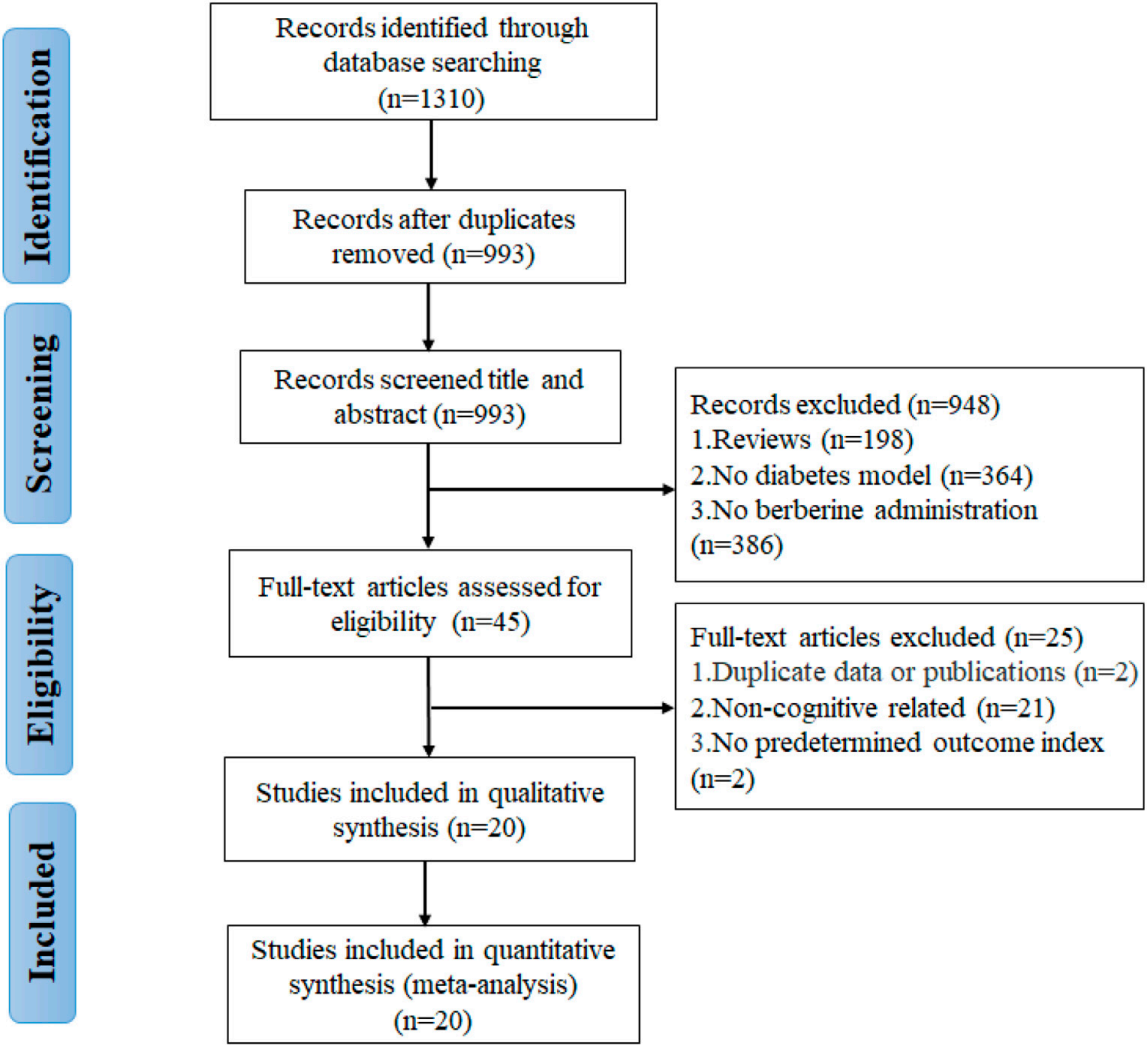
Flowchart of selection for studies inclusion.

A total of 1,310 potentially relevant articles were found through database searching, including 283 from PubMed, 97 from Web of Science, 179 from Embase, 279 from CNKI, 284 from Wanfang, and 188 from VIP. After incorporating all searches and removing duplicates, 993 records are retained. Among the remaining articles, 948 records were eliminated by reading the titles and abstracts. Finally, through the full-text assessment, 20 studies met the eligibility requirements and were included in this systematic review and meta-analysis (Figure 2). All included studies were published during the past decade (2011–2021), indicating that the neuroprotective effects of berberine on DCI have attracted intense interest in recent years.

### 3.2 Characteristics of Included Studies

In the 20 included studies, 13 studies were published in English (Bhutada et al., 2011; Kalalian-Moghaddam et al., 2013; Moghaddam et al., 2013; Moghaddam et al., 2014; Zhou et al., 2016; Chen et al., 2017; Li et al., 2018; Wang et al., 2018; Wang et al., 2019; Adefegha et al., 2021; Tian et al., 2021; Wu et al., 2021; Zhang et al., 2021) and 7 studies were published in Chinese (Ma et al., 2012a; Ma et al., 2012b; Tong et al., 2019; Cao et al., 2021; Liu, 2021; Ma and Liu, 2021; Xuan, 2021). A total of 442 diabetic model animals were enrolled, including 278 in the treatment group and 164 in the control group. Ten studies used Wistar rats (268/442, 60.6%), four used Sprague Dawley rats (82/442, 18.6%), two used db/db mice (32/442, 7.2%), two used ICR mice (60/442, 13.6%), one used the Kunming mice (36/442, 8.1%), and one did not specify which species of rats were used. All the animal models included in the studies were rats or mice. Seventeen studies used male animals, two used female animals, and one did not specify the sex of the animals. The initial weight of animals was 170–220 g in 11 studies, 200–230 g in 1 study, 225–285 g in 3 studies, 20–30 g in 2 studies, and not mentioned in 3 studies. Only eight studies reported the age of animals, ranging from 4 to 10 weeks. In the preparation of diabetes models, seven studies established animal models by intraperitoneal injection of streptozotocin (STZ) only, and the dose range for STZ injections was 40–60 mg/kg (Bhutada et al., 2011; Kalalian-Moghaddam et al., 2013; Moghaddam et al., 2013; Moghaddam et al., 2014; Tong et al., 2019; Adefegha et al., 2021; Ma and Liu, 2021). Six studies were to induced diabetes models by intraperitoneal injection of STZ combined with a high-sugar and high-fat diet (HSFD) or high-fat diet (HFD), and the dose range of STZ was 30–50 mg/kg (Ma et al., 2012a; Ma et al., 2012b; Zhou et al., 2016; Liu, 2021; Xuan, 2021; Zhang et al., 2021). Five studies were to induce diabetes models by caudal vein injection of STZ (25 mg/kg) combined with HSFD or HFD (Chen et al., 2017; Wang et al., 2018; Wang et al., 2019; Tian et al., 2021; Wu et al., 2021). Two studies were spontaneous diabetes models (Li et al., 2018; Cao et al., 2021). All studies used FBG > 11.1 mmol/L or 2hBG > 11.1 mmol/L of OGTT as the standard to evaluate model success. The maximum duration of administration was 23 weeks (range, 2–23 weeks), and the maximum dose of berberine was 200 mg/kg/d (range, 20–200 mg/kg/d). In order to assess the effect of berberine on blood glucose in diabetic models, 15 studies recorded the FBG level (Bhutada et al., 2011; Ma et al., 2012a; Ma et al., 2012b; Kalalian-Moghaddam et al., 2013; Moghaddam et al., 2013; Moghaddam et al., 2014; Zhou et al., 2016; Wang et al., 2018; Tong et al., 2019; Wang et al., 2019; Adefegha et al., 2021; Cao et al., 2021; Liu, 2021; Tian et al., 2021; Xuan, 2021), 3 studies recorded serum insulin (Li et al., 2018; Wang et al., 2018; Wang et al., 2019), 3 studies recorded the OGTT level (Wang et al., 2019; Cao et al., 2021; Zhang et al., 2021), 2 studies recorded the ITT level (Li et al., 2018; Cao et al., 2021), and 1 study recorded the HbA1c level (Zhou et al., 2016). Behavioral tests were used to evaluate the learning and memory function of animals. For further assessment of the neuroprotective effect of berberine on DCI model animals, 14 studies conducted the MWM test (Bhutada et al., 2011; Ma et al., 2012b; Zhou et al., 2016; Li et al., 2018; Wang et al., 2018; Tong et al., 2019; Wang et al., 2019; Cao et al., 2021; Liu, 2021; Ma and Liu, 2021; Tian et al., 2021; Wu et al., 2021; Xuan, 2021; Zhang et al., 2021), 2 studies conducted the Y-maze test (Kalalian-Moghaddam et al., 2013; Li et al., 2018), 2 studies conducted the conditioned fear test (Chen et al., 2017; Li et al., 2018), and 1 study conducted the passive avoidance test (Kalalian-Moghaddam et al., 2013). Detailed information on berberine in each study is displayed in Table 1. The characteristics and details of the included studies are presented in Table 2.

**TABLE 1:** Information of berberine of each study.

Study (years)	Source	Purity (%)	Quality control reported
Bhutada et al. (2011)	Sami Labs (Bangalore, India)	>97%	Unknown
Ma et al. (2012a)	Nanjing Zelang Pharmaceutical Technology Co., Ltd. (Nanjing, China)	>97%	Y-HPLC
Ma et al. (2012b)	Nanjing Zelang Pharmaceutical Technology Co., Ltd. (Nanjing, China)	>97%	Y-HPLC
Kalalian-Moghaddam et al. (2013)	Sigma-Aldrich (St. Louis, MO, United States)	>95%	Y-HPLC
Moghaddam et al. (2013)	Sigma-Aldrich (St. Louis, MO, United States)	>95%	Y-HPLC
Moghaddam et al. (2014)	Sigma-Aldrich (St. Louis, MO, United States)	>95%	Y-HPLC
Zhou et al. (2016)	Northeast Pharmaceutical Group (Shenyang, China)	>98%	Y-HPLC
Chen et al. (2017)	Sigma-Aldrich (St. Louis, MO, United States)	>95%	Y-HPLC
Wang et al. (2018)	Sigma-Aldrich (St. Louis, MO, United States)	>95%	Y-HPLC
Li et al. (2018)	Sigma-Aldrich (St. Louis, MO, United States)	>95%	Y-HPLC
Wang et al. (2019)	Shanghai Yuanye Bio-Technology Co., Ltd. (Shanghai, China)	≥98%	Y-HPLC
Tong et al. (2019)	Unknown	Unknown	Unknown
Xuan (2021)	Sigma-Aldrich (St. Louis, MO, United States)	>95%	Y-HPLC
Liu (2021)	Sigma-Aldrich (St. Louis, MO, United States)	>95%	Y-HPLC
Adefegha et al. (2021)	Sigma-Aldrich (St. Louis, MO, United States)	>95%	Y-HPLC
Zhang et al. (2021)	Northeast Pharmaceutical Group (Shenyang, China)	>98%	Y-HPLC
Wu et al. (2021)	Sigma-Aldrich (St. Louis, MO, United States)	>95%	Y-HPLC
Cao et al. (2021)	Northeast Pharmaceutical Group (Shenyang, China)	>98%	Y-HPLC
Tian et al., 2021	Unknown	Unknown	Unknown
Ma and Liu (2021)	Shanghai Yuanye Bio-Technology Co., Ltd. (Shanghai, China)	≥98%	Y-HPLC

**TABLE 2:** Basic characteristics of the 20 included studies.

Study (year)	Species (sex, *n* = treatment/control group, age, weight)	Modeling method and standard	Berberine intervention (administration, drug dose, duration)	Outcomes	Intergroup difference
Bhutada et al. (2011)	Wistar rats (male, 18/6, 200–220 g)	Intraperitoneal injection of STZ (60 mg/kg),	By intragastric	1. FBG	1. *p* < 0.01
		BG > 250 mg/dl	25/50/100 mg/kg/d	2. MWM	2. *p* < 0.05
			4 weeks	3. LPO	3. *p* < 0.01
				4. GSH	4. *p* < 0.01
				5. AChE	5. *p* < 0.01
Ma et al. (2012a)	Sprague-Dawley rats (male, 18/6, 180–220 g)	Intraperitoneal injection of STZ (40 mg/kg) + HSFD	By intragastric	1. FBG	1. *p* < 0.01
		BG > 11.1 mmol/L	50/100/200 mg/kg/d	2. MWM	2. *p* < 0.05
			4 weeks	3. SOD, MDA	3. *p* < 0.05
				4. AChE	4. *p* < 0.01
				5. Bax, Bcl-2	
				6. IGF-1	
Ma et al. (2012b)	Sprague-Dawley rats (male, 18/6, 180–220 g)	Intraperitoneal injection of STZ (40 mg/kg) + HSFD	By intragastric	1. FBG	1. *p* < 0.05
		BG ≥ 16.7 mmol/L	50/100/200 mg/kg/d	2. CAT	2. *p* < 0.01
			4 weeks	3. LPO	3. *p* < 0.01
				4. GSH	4. *p* < 0.01
				5. BDNF	
Kalalian-Moghaddam et al. (2013)	Wistar rats (male, 12/6, 225–285 g)	Intraperitoneal injection of STZ (55 mg/kg)	By intragastric	1. FBG	1. *p* < 0.01
		BG > 250 mg/dl	50/100 mg/kg/d	2. Y-maze	2. *p* < 0.05
			11 weeks	3. Passive avoidance	3. *p* < 0.05
				4. PS, EPSP	4. *p* < 0.01
				5. Nissl/TUNEL neuron	5.*p* < 0.05
Moghaddam et al. (2013)	Wistar rats (male, 20/10, 225–285 g)	Intraperitoneal injection of STZ (55 mg/kg),	By intragastric	1. FBG	1. *p* < 0.01
		BG > 250 mg/dl	50/100 mg/kg/d	2. PS, EPSP	2. *p* < 0.01
			12 weeks		
Moghaddam et al. (2014)	Wistar rats (male, 24/12, 225–285 g)	Intraperitoneal injection of STZ (55 mg/kg),	By intragastric	1. FBG	1. *p* < 0.05
		BG > 250 mg/dl	50/100 mg/kg/d	2. MDA	2. *p* < 0.05
			8 weeks	3. SOD	3. *p* < 0.05
				4. NO	4. *p* < 0.05
				5. GFAP	5. *p* < 0.05
Zhou et al. (2016)	Wistar rats (male, 10/10, 180–220 g)	Intraperitoneal injection of STZ (35 mg/kg) + HSFD	By intragastric	1. FBG	1. *p* < 0.01
		BG > 16.7 mmol/L	100 mg/kg/d	2. HbA1c	2. *p* < 0.01
			23 weeks	3. MWM	3. *p* < 0.01
				4. TG, TC	4. *p* < 0.01
				5. p-p38/p38 6. p-JNK/JNK 7.p-ERK/ERK	5. *p* < 0.01
					6. *p* < 0.01
					7. *p* > 0.05
Chen et al. (2017)	Wistar rats (male, 8/8, aged 4–5 weeks, about 200 g)	Caudal vein injection of STZ (25 mg/kg) + HSFD	By intragastric	1. Conditioned fear	1. *p* < 0.01
		BG ≥ 11.2 mmol/L	187.75 mg/kg/d	2. PDG PET	2. *p* < 0.01
			10 weeks	3. p-IRS-1	3. *p* < 0.01
				4. p-PI3K/PI3K	4. *p* < 0.05
				5. p-Akt/Akt	5. *p* < 0.05
				6. p-GSK3β/GSK3β 7. p-IKKβ, p-NF-κB/NF-κB 8. p-JNK/JNK	6. *p* < 0.05
				9. IL-1β, TNF-α	7. *p* < 0.05
				10. GLUT3	8. *p* < 0.05
				11. PKC	9. *p* > 0.05
				12. APP	10. *p* < 0.01
				13. BACE1	11. *p* < 0.05
					12. *p* < 0.01
					13. *p* < 0.05
Wang et al. (2018)	Wistar rats (male, 15/12, aged 5–6 weeks, 180–200 g)	Caudal vein injection of STZ (25 mg/kg) + HFD,	By intragastric	1. FBG	1. *p* < 0.05
		BG ≥ 11.2 mmol/L	100/200 mg/kg/d	2. Serum insulin	2. *p* < 0.05
			10 weeks	3. MWM	3. *p* < 0.01
				4. p-Tau Ser202/Tau-5	4. *p* < 0.01
				5. p-Tau Ser404/Tau-5 6. p-PI3K/PI3K	5. *p* < 0.01
				7. p-Akt/Ak	6. *p* < 0.01
				8. p-GSK3β/GSK3β	7. *p* < 0.01
					8. *p* < 0.01
Li et al. (2018)	db/db mice (female, 8/8,7 weeks)	Spontaneous diabetic model	By intragastric	1. Serum insulin	1. *p* < 0.05
			50 mg/kg/d	2. 2hBG of OGTT	2. *p* > 0.05
			10 weeks	3. 2hBG of ITT	3. *p* < 0.05
				4. MWM	4. *p* < 0.05
				5. Y-maze, conditioned fear	5. *p* < 0.01
				6. TG, TC, HDL-C	6. *p* < 0.05
				7. LDL-C	7. *p* < 0.01
				8. PSD95, SYN, NGF	8. *p* < 0.01
				9. TNF-α, NF-κB	9. *p* < 0.05
				10. SIRT1 11. p-PERK/PERK	10. *p* < 0.05
				12. p-IRE1α/IRE1α	11. *p* < 0.05
				13. p-eIF2α/eIF2α	12. *p* < 0.05
				14. CHOP	13. *p* < 0.05
					14. *p* < 0.05
Wang et al. (2019)	Wistar rats (male, 8/8, aged 4–5 weeks, 180–200 g)	Caudal vein injection of STZ (25 mg/kg) + HSFD,	By intragastric	1. FBG	1. *p* < 0.01
		BG > 11.2 mmol/L	187.75 mg/kg/d	2. Serum insulin	2. *p* < 0.01
			10 weeks	3. MWM	3. *p* < 0.01
				4. PDG PET	4. *p* < 0.01
				5. IL-18, IL-1β	5. *p* < 0.05
				6. TNF-α 7. p-IKK/IKK 8. p-NF-κB/NF-κB	6. *p* < 0.01
				9. GFAP	7. *p* < 0.05
				10. AChE	8. *p* < 0.05
				11. Insulin receptor, p-IRS1	9. *p* < 0.01
				12. PI3K, p-Akt/Akt	10. *p* < 0.05
				13. APP, BACE1	11. *p* < 0.05
				14. Aβ42	12. *p* < 0.01
					13. *p* < 0.01
					14. *p* < 0.05
Tong et al. (2019)	Sprague-Dawley rats (male, 9/9, 170–200 g)	Intraperitoneal injection of STZ (55 mg/kg),	By intragastric	1. FBG	1. *p* > 0.05
		BG > 16.7 mmol/L	60 mg/kg/d	2. MWM	2. *p* < 0.05
			6 weeks	3. Bcl-2, Caspase-3	3. *p* < 0.05
				4. Notch1	4. *p* < 0.05
				5. TUNEL neuron	5. *p* < 0.01
				6. Brain pathology	
Xuan (2021)	ICR mice (male, 15/15, aged 8 weeks)	Intraperitoneal injection of STZ (50 mg/kg) + HSFD,	By intragastric	1. FBG	1. *p* < 0.05
		BG ≥ 13.9 mmol/L	50 mg/kg/d	2. MWM	2. *p* < 0.05
			8 weeks	3. Nissl neuron	3. *p* < 0.01
				4. MDA	4. *p* < 0.01
				5. Bcl-2, Bax	5. *p* < 0.01
				6. Caspase-3	6. *p* < 0.01
				7. Nrf2, HO-1	7. *p* < 0.01
				8. NQO1	8. *p* < 0.01
Liu (2021)	ICR mice (male, 15/15, aged 8 weeks, 25–30 g)	Intraperitoneal injection of STZ (50 mg/kg) + HSFD,	By intragastric	1. FBG	1. *p* < 0.05
		BG ≥ 13.9 mmol/L	50 mg/kg/d	2. MWM	2. *p* < 0.05
			8 weeks	3. Nissl neuron	3. *p* < 0.05
				4. MDA	4. *p* < 0.05
				5. NF-κB	5. *p* < 0.01
				6. TNF-α	6. *p* < 0.05
				7. IL-1β	7. *p* < 0.01
				8. Nrf2, HO-1	8. *p* < 0.01
				9. NQO1	9. *p* < 0.05
Adefegha et al. (2021)	Rats (16/8, 200–230 g)	Intraperitoneal injection of STZ (50 mg/kg)	By intragastric	1. FBG	1. *p* < 0.05
		BG ≥ 250 mg/dl	50/100 mg/kg/d	2. AChE	2. *p* < 0.01
			2 weeks	3. BChE	3. *p* < 0.05
				4. MAO, MDA	4. *p* < 0.01
				5. SOD, GSH-Px	5. *p* < 0.05
				6. GSH	6. *p* > 0.05
Zhang et al. (2021)	Sprague-Dawley rats (male, 8/8, 180–220 g)	Intraperitoneal injection of STZ (30 mg/kg) + HFD	By intragastric	1. 2hBG of OGTT	1. *p* < 0.01
		BG > 16.7 mmol/L	150 mg/kg/d	2. MWM	2. *p* < 0.01
			4 weeks	3. Aβ	3. *p* < 0.05
				4. Tau	4. *p* < 0.01
				5. p-Tau	5. *p* < 0.05
				6. Insulin receptor	6. *p* < 0.01
				7. Nissl/TUNEL neuron	7. *p* < 0.01
Wu et al. (2021)	Wistar rats (male, aged 6–8 weeks, 180–200 g)	Caudal vein injection of STZ (25 mg/kg) + HSFD	By intragastric	1. MWM	1. *p* <0.05
		BG > 11.2 mmol/L	200 mg/kg/d	2. p-IRS-1	
			13 weeks	3. PI3K, PKC	
				4. GLUT3	
Cao et al. (2021)	db/db mice (female, 8/8, aged 10 weeks)	Spontaneous diabetic model	By intragastric	1. FBG	1. *p* < 0.01
			135 mg/kg/d	2. 2hBG of OGTT	2. *p* < 0.01
			10 weeks	3. 2hBG of ITT	3. *p* < 0.01
				4. HbA1c	4. *p* < 0.01
				5. MWM	5. *p* < 0.05
				6. TNF-α	6. *p* < 0.01
				7. IL-1β, IL-6	7. *p* < 0.01
				8. NLRP3	8. *p* < 0.01
Tian et al., 2021	Wistar rats (male, 8/8, aged 6–8 weeks, 180–200 g)	caudal vein injection of STZ (25 mg/kg) + HSFD	By intragastric	1. FBG	1. *p* < 0.01
		BG > 11.2 mmol/L	50/200 mg/kg/d	2. MWM	2. *p* < 0.01
			4 weeks	3. MMP	3. *p* < 0.01
				4. ROS, GSH, MDA	4. *p* < 0.01
				5. RhoC, ROCK	5. *p* < 0.01
Ma and Liu et al. (2021)	Kunming mice (male, 27/10, 20–25 g)	Intraperitoneal injection of STZ (40 mg/kg)	By intragastric	1. MWM	1. *p* < 0.05
		BG > 11.1 mmol/L	20/40/80 mg/kg/d	2. TNF-α	2. *p* < 0.05
			4 weeks	3. IL-1β	3. *p* < 0.05
				4. AGEs	4. *p* < 0.05
				5. RAGE	5. *p* < 0.05
				6. NF-κB	6. *p* < 0.01

AChE, acetylcholinesterase; AGEs, advanced glycation end products; Akt, protein kinase B; APP, amyloid precursor protein; Aβ, amyloid β; BACE1, beta-secretase 1; BChE, butyrylcholinesterase; Bcl-2, B-cell lymphoma-2; Bax, bcl2-associated x; BDNF, brain-derived neurotrophic factor; CAT, catalase; CHOP, C/EBP homologous protein; eIF2α, eukaryotic initiation factor 2α; EPSP, excitatory post-synaptic potential; EPK, extracellular-regulated protein kinases; BG, blood glucose; FBG, fasting blood glucose; FDG PET, fluorodeoxyglucose positron emission tomography; GFAP, glial fibrillary acidic protein; GLUT3, glucose transporter 3; GSH, glutathione; GSH-Px, glutathione peroxidase; GSK3β, glycogen synthase kinase 3β; HbA1c, hemoglobin A1c; HDL-C, high-density lipoprotein cholesterol; HO-1, haeme oxygenase-1; HFD, high-fat diet; HSFD, high-sugar and high fat diet; IGF-1, insulin-like growth factor 1; IKK, inhibitor of kappa B kinase; IL-1β, interleukin-1β; IRE1α, inositol-requiring enzyme 1α; IRS, insulin receptor substrate; ITT, insulin tolerance test; JNK, c-Jun N-terminal kinases; LDL-C, low-density lipoprotein cholesterol; LPO, lipid peroxidation; MAO, monoamine oxidase; MDA, malondialdehyde; MWM, Morris water maze; MMP, matrix metalloproteinase; NF-κB, nuclear factor-κB; NGF, nerve growth factor; NO, nitric oxide; NQO1, NADPH quinone oxidoreductase 1; Nrf2, nuclear factor erythroid-2 related factor 2; OGTT, oral glucose tolerance test; PERK, protein kinase R-like ER kinase; PI3K, phosphatidylinositide 3-kinases; PKC, protein kinase C; PS, population spike; PSD95, postsynapticdensity 95; RAGE, receptors for advanced glycation end products; ROCK, Rho-associated kinase; ROS, reactive oxygen species; SIRT1, sirtuin1; SOD, superoxide dismutase; STZ, streptozotocin; SYN, synaptophysin; TC, total cholesterol; TG, triglyceride; TNF-α, tumor necrosis factor α.

### 3.3 Quality of Included Studies

We evaluated the quality of all the included studies. The scores ranged from 3 to 5 for each study, and the overall quality was low. Four studies got 5 points (Chen et al., 2017; Li et al., 2018; Wang et al., 2018; Cao et al., 2021), nine studies got 4 points (Kalalian-Moghaddam et al., 2013; Moghaddam et al., 2013; Tong et al., 2019; Wang et al., 2019; Adefegha et al., 2021; Liu, 2021; Tian et al., 2021; Xuan, 2021; Zhang et al., 2021), and seven studies got 3 points (Bhutada et al., 2011; Ma et al., 2012a; Ma et al., 2012b; Moghaddam et al., 2014; Zhou et al., 2016; Ma and Liu, 2021; Wu et al., 2021). Among the 20 included studies, only two studies reported random sequence generation (Liu, 2021; Xuan, 2021), and nine studies fully reported the baseline characteristics (Kalalian-Moghaddam et al., 2013; Moghaddam et al., 2013; Moghaddam et al., 2014; Chen et al., 2017; Li et al., 2018; Wang et al., 2018; Wang et al., 2019; Cao et al., 2021; Tian et al., 2021), and nine studies described randomness of housing (Chen et al., 2017; Li et al., 2018; Wang et al., 2018; Tong et al., 2019; Wang et al., 2019; Adefegha et al., 2021; Cao et al., 2021; Liu, 2021; Zhang et al., 2021). Allocation concealment, blind intervention, random for outcome evaluation, and blinding for outcomes were not reported in any studies. The methodological quality of the included studies is displayed in Table 3.

**TABLE 3:** The methodological quality of included studies.

Study	A	B	C	D	E	F	G	H	I	J	Total
Bhutada et al. (2011)	−	?	−	?	−	−	−	+	+	+	3
Ma et al. (2012a)	−	?	−	?	−	−	−	+	+	+	3
Ma et al. (2012b)	−	?	−	?	−	−	−	+	+	+	3
Kalalian-Moghaddam et al. (2013)	−	+	−	?	−	−	−	+	+	+	4
Moghaddam et al. (2013)	−	+	−	?	−	−	−	+	+	+	4
Moghaddam et al. (2014)	−	+	−	?	−	−	−	−	+	+	3
Zhou et al. (2016)	−	?	−	?	−	−	−	+	+	+	3
Chen et al. (2017)	−	+	−	+	−	−	−	+	+	+	5
Wang et al. (2018)	−	+	−	+	−	−	−	+	+	+	5
Li et al. (2018)	−	+	−	+	−	−	−	+	+	+	5
Wang et al. (2019)	−	+	−	+	−	−	−	−	+	+	4
Tong et al. (2019)	−	?	−	+	−	−	−	+	+	+	4
Xuan (2021)	+	−	−	?	−	−	−	+	+	+	4
Liu (2021)	+	−	−	+	−	−	−	+	+	+	4
Adefegha et al. (2021)	−	?	−	+	−	−	−	+	+	+	4
Zhang et al. (2021)	−	?	−	+	−	−	−	+	+	+	4
Wu et al. (2021)	−	?	−	?	−	−	−	+	+	+	3
Cao et al. (2021)	−	+	−	+	−	−	−	+	+	+	5
Tian et al., 2021	−	+	−	?	−	−	−	+	+	+	4
Ma and Liu (2021)	−	?	−	?	−	−	−	+	+	+	3

A, sequence generation; B, baseline characteristics; C, allocation concealment; D, random housing; E, blinding (caregivers/investigators); F, random for outcome assessment; G, blinding (outcome assessor); H, incomplete outcome data; I, selective outcome reporting; J, other biases. +, low-risk of bias; −, high-risk of bias; ?, unclear-risk of bias.

### 3.4 Effectiveness

#### 3.4.1 Primary Outcomes

##### 3.4.1.1 Effect of Berberine on Fasting Blood Glucose

**FIGURE 3:**
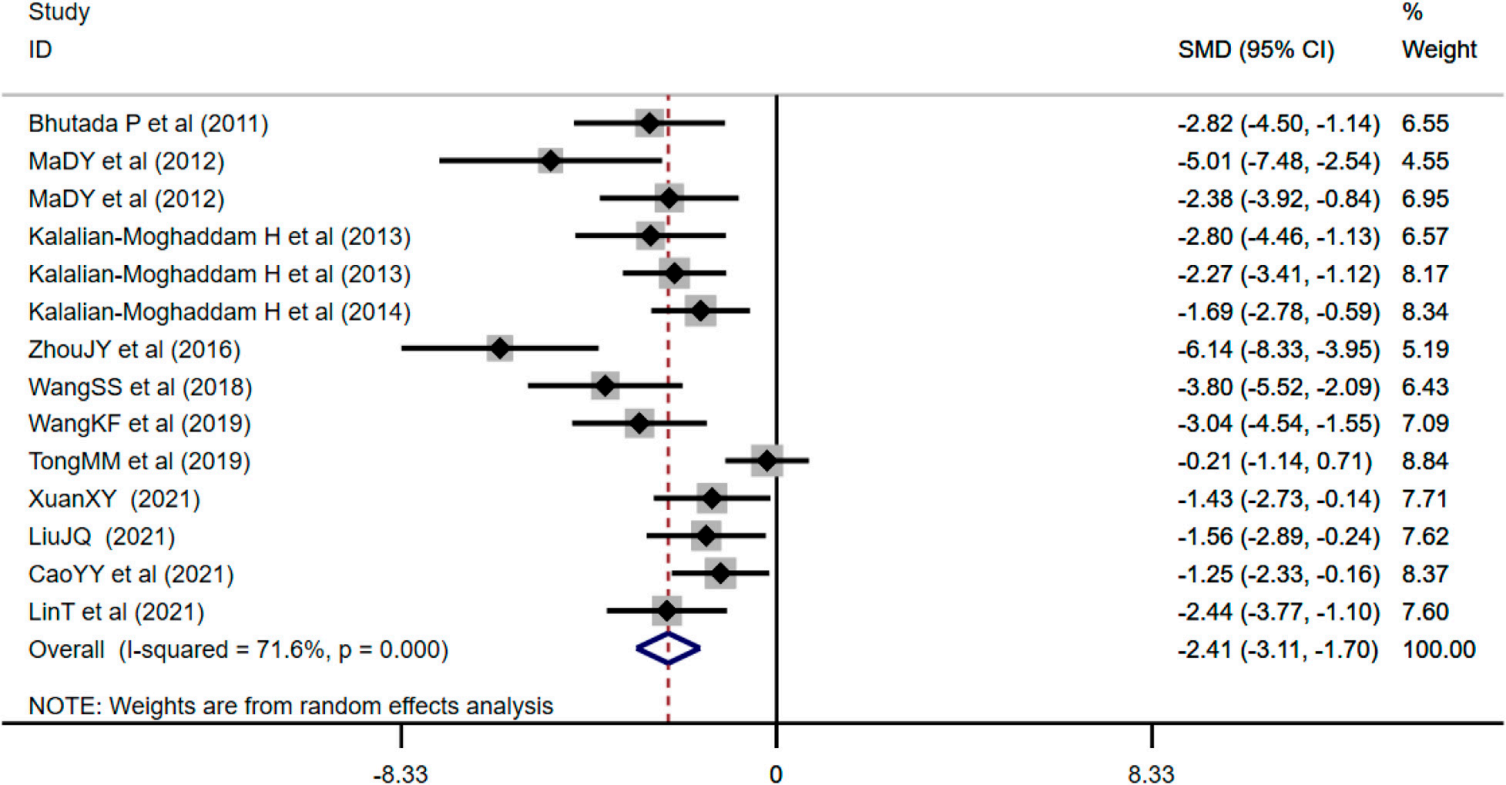
Forest plot: effect of berberine on FBG level.

FBG was adopted as the outcome measure in 14 studies and used random effects analysis combined with an effect size. The overall outcome showed berberine could reduce FBG level compared with the control group (*n* = 212; SMD: −2.41 [95% CI: −3.11, −1.70], *p* < 0.001; heterogeneity: χ2 = 45.84, df = 13, *p* < 0.001, *I* ^2^ = 71.6%, Figure 3).

##### 3.4.1.2 Effect of Berberine on Morris Water Maze Test

**FIGURE 4:**
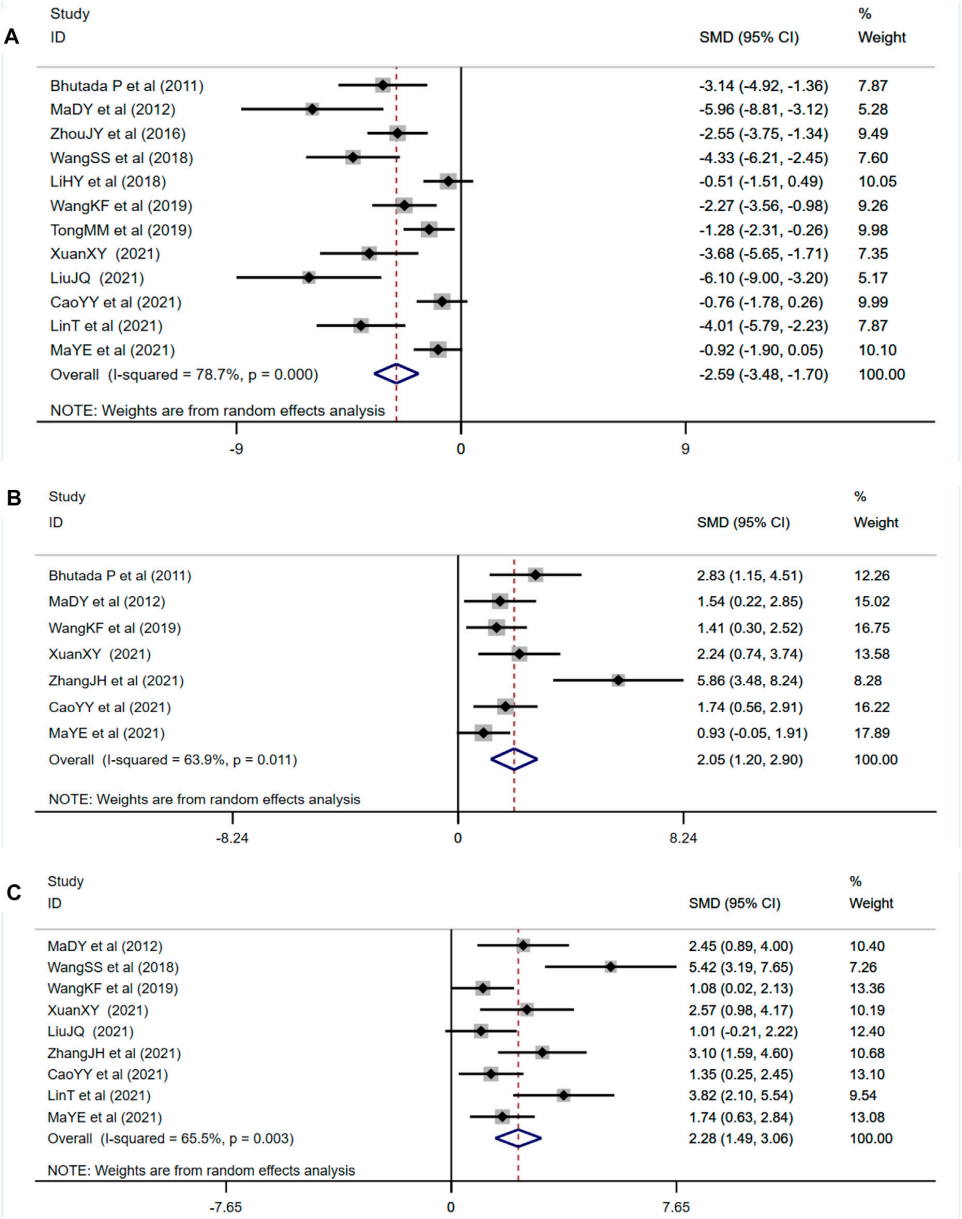
Forest plot: effect of berberine on **(A)** escape latency, **(B)** times of crossing the platform, and **(C)** time spent in the target quadrant.

As one of the primary outcome measures, the MWM could reflect changes in learning memory of diabetic animals. Among the 14 studies included, 12 studies reported the escape latency, 7 studies reported times of platform crossing, and 9 studies reported the time spent in the target quadrant. Compared with the control group, berberine treatment group could significantly shorten escape latency (*n* = 184; SMD: −2.59 [95% CI: −3.48, −1.70], *p* < 0.001; heterogeneity: χ^2^ = 51.53, df = 11, *p* < 0.001, I^2^ = 78.7%, Figure 4A). Compared with the control group, the times of crossing the platform increased in the berberine treatment group (*n* = 102; SMD: 2.05 [95% CI: 1.20, 2.90], *p* < 0.001; heterogeneity: χ^2^ = 16.61, df = 6, *p* = 0.011, I^2^ = 63.9%, Figure 4B). Compared with the control group, the time spent in the target quadrant increased in the berberine treatment group (*n* = 134; SMD: 2.28 [95% CI: 1.49, 3.06], *p* < 0.001; heterogeneity: χ^2^ = 23.17, df = 8, *p* = 0.003, I^2^ = 65.5%, Figure 4C).

#### 3.4.2 Secondary Outcomes

##### 3.4.2.1 Effect of Berberine on Serum Insulin

**FIGURE 5:**
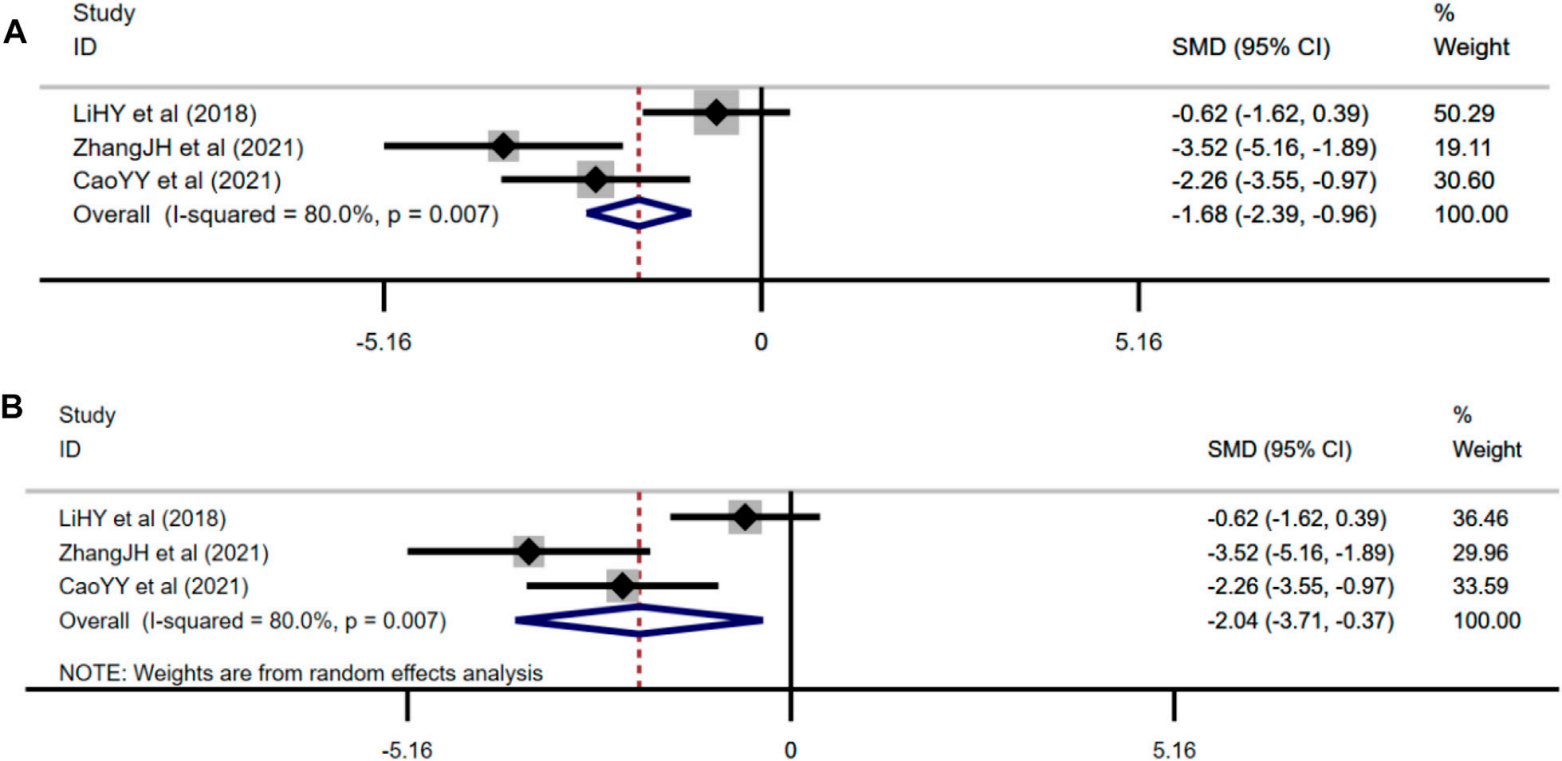
Forest plot: effect of berberine on **(A)** serum insulin and **(B)** 2hBG of OGTT.

Three studies adopted serum insulin as one of the outcome measures and used fixed effects model combined with an effect size. The results indicated berberine could reduce serum insulin level compared with the control group (*n* = 48; SMD: −2.14 [95% CI: −2.88, −1.40], *p* < 0.001; heterogeneity: χ^2^ = 2.52, df = 2, *p* = 0.284, I^2^ = 20.6%, Figure 5A).

##### 3.4.2.2 Effect of Berberine on 2hBG of Oral Glucose Tolerance Test

Three studies used 2hBG of OGTT as one of the outcome measures and used random effects analysis combined with an effect size. The overall outcome showed berberine could reduce 2hBG of the OGTT level compared with the control group (*n* = 48; SMD: −2.04 [95% CI: −3.71, −0.37], *p* = 0.016; heterogeneity: χ^2^ = 10, df = 2, *p* = 0.007, I^2^ = 80%, Figure 5B).

##### 3.4.2.3 Effect of Berberine on Amyloid β

**FIGURE 6:**
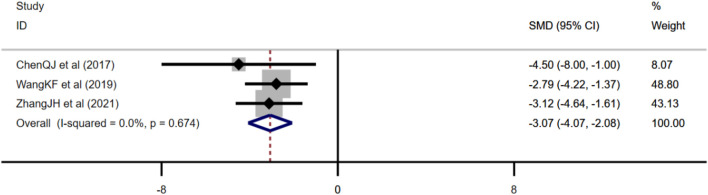
Forest plot: effect of berberine on Aβ.

Three studies adopted Aβ as an outcome measure and used fixed effects model combined with an effect size. The results indicated berberine could reduce the Aβ level compared with the control group (*n* = 38; SMD: −3.07 [95% CI: −4.07, −2.08], *p* < 0.001; heterogeneity: χ^2^ = 0.79, df = 2, *p* = 0.674, I^2^ = 0, Figure 6).

##### 3.4.2.4 Effect of Berberine on Oxidative Stress Index

**FIGURE 7:**
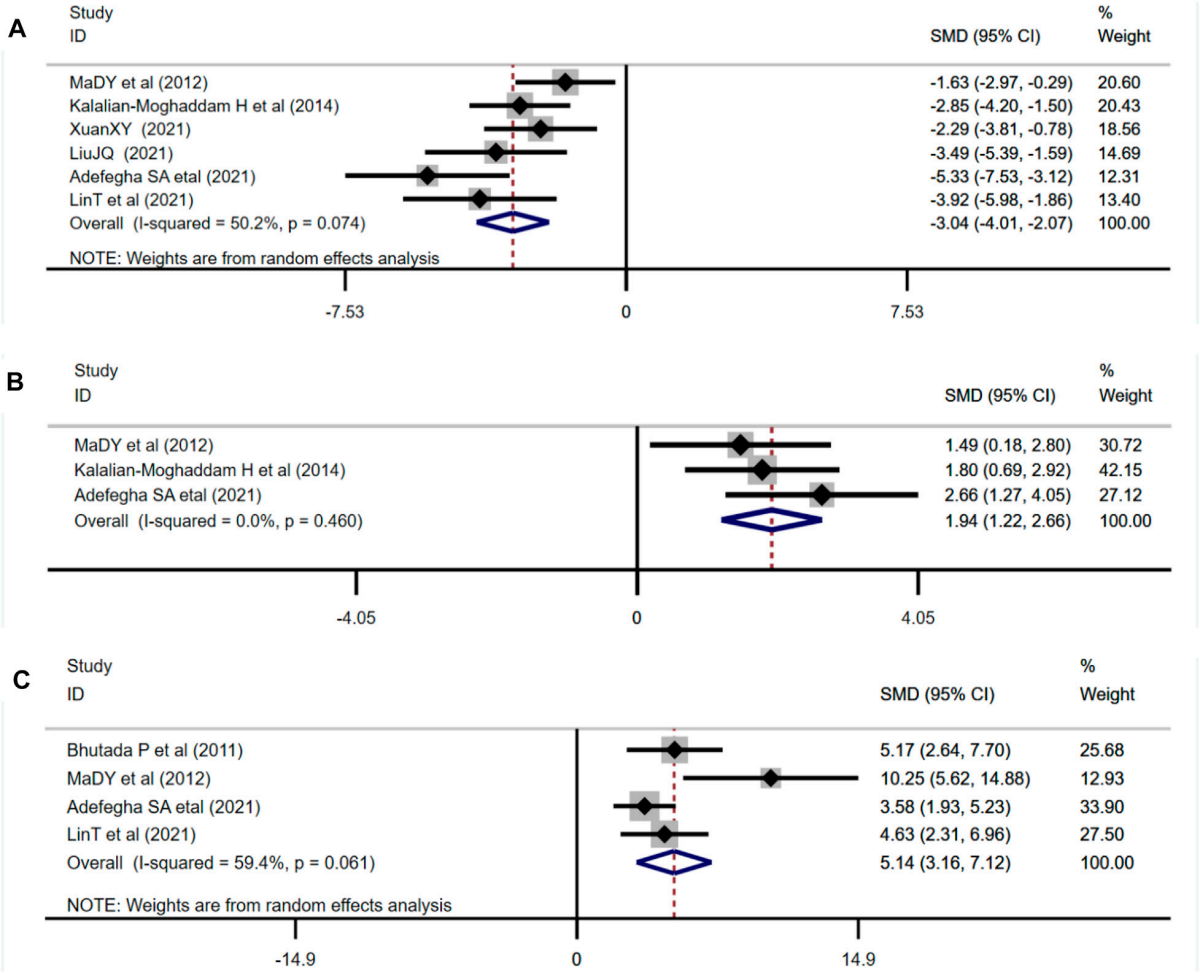
Forest plot: effect of berberine on **(A)** MDA, **(B)** SOD, and **(C)** GSH.

Six studies adopted MDA as an outcome measure and used random effects analysis combined with an effect size. The results indicated berberine could reduce the MDA level compared with the control group (*n* = 82; SMD: −3.04 [95% CI: −4.01, −2.07], *p* < 0.001; heterogeneity: χ^2^ = 10.05, df = 5, *p* = 0.074, I^2^ = 50.2%, Figure 7A). Three studies adopted SOD as an outcome measure and used fixed effects model combined with an effect size. The results indicated berberine could increase SOD level compared with the control group (*n* = 46; SMD: 1.94 [95% CI: 1.22, 2.66], *p* < 0.001; heterogeneity: χ^2^ = 1.55, df = 2, *p* = 0.460, I^2^ = 0, Figure 7B). Four studies adopted GSH as an outcome measure and used random effects analysis combined with an effect size. The results indicated berberine could increase GSH level compared with the control group (*n* = 52; SMD: 5.14 [95% CI: 3,16, 7.12], *p* < 0.001; heterogeneity: χ^2^ = 7.38, df = 3, *p* = 0.061, I^2^ = 59.4%, Figure 7C).

##### 3.4.2.5 Effect of Berberine on Inflammation Levels

**FIGURE 8:**
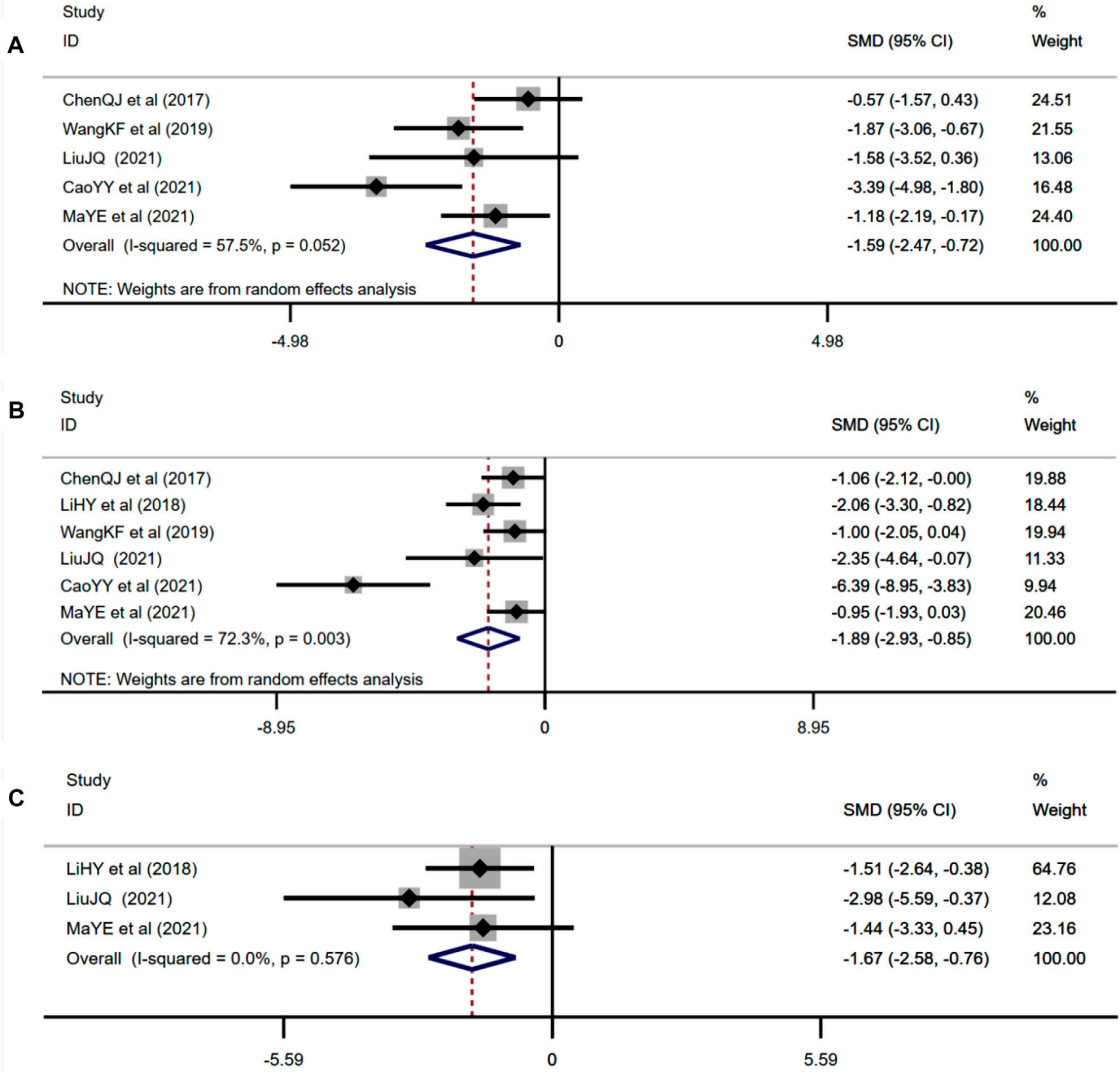
Forest plot: effect of berberine on **(A)** IL-1β, **(B)** TNF-α, and **(C)** NF-κB.

Five studies adopted IL-1β as an outcome measure and used random effects analysis combined with an effect size. The results indicated berberine could reduce the IL-1β level compared with the control group (*n* = 72; SMD: −1.59 [95% CI: −2.47, −0.72], *p* < 0.001; heterogeneity: χ^2^ = 9.41, df = 4, *p* = 0.052, I^2^ = 57.5%, Figure 8A). Six studies adopted TNF-α as an outcome measure and used random effects analysis combined with an effect size. The results indicated berberine could reduce the TNF-α level compared with the control group (*n* = 88; SMD: −1.89 [95% CI: −2.93, −0.85], *p* < 0.001; heterogeneity: χ^2^ = 18.02, df = 5, *p* = 0.003, I^2^ = 72.3%, Figure 8B). Three studies adopted NF-κB as an outcome measure and used the fixed effects model combined with an effect size. The results indicated berberine could reduce the NF-κB level compared with the control group (*n* = 28; SMD: −1.67 [95% CI: −2.58, −0.76], *p* < 0.001; heterogeneity: χ^2^ = 1.10, df = 2, *p* = 0.576, I^2^ = 0, Figure 8C).

##### 3.4.2.6 Effect of Berberine on Acetylcholinesterase

**FIGURE 9:**
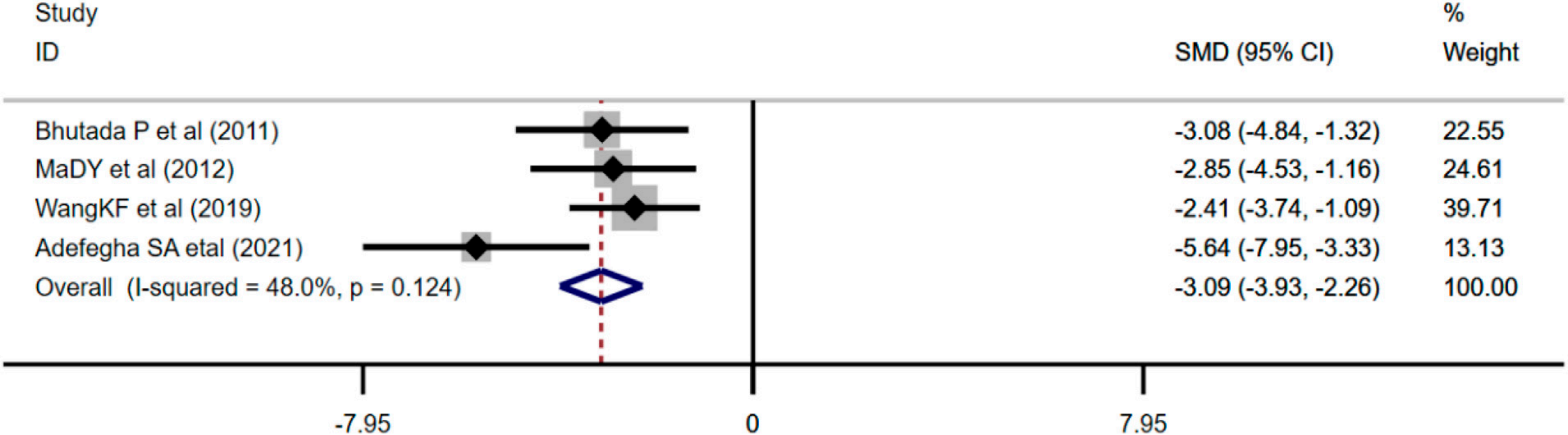
Forest plot: effect of berberine on AChE.

Four studies adopted AChE as an outcome measure and used the fixed effects model combined with an effect size. The results indicated berberine could reduce the AChE level compared with the control group (*n* = 76; SMD: −3.09 [95% CI: −3.93, −2.26], *p* < 0.001; heterogeneity: χ^2^ = 5.76, df = 3, *p* = 0.124, I^2^ = 48%, Figure 9).

### 3.5 Subgroup Analysis

Due to the high heterogeneity among the studies, we evaluated five subgroups for FBG and escape latency depending on the year of publication, animal species, the dose of STZ, the dose of berberine, and the duration of treatment. The results showed that animal species and year of publication could be the source of heterogeneity for FBG. For escape latency, the subgroup analysis based on year of publication, animal species, dose of STZ, dose of berberine, and duration of treatment did not reveal the sources of heterogeneity among the studies. There was still significant heterogeneity among studies. The results are presented in Table 4.

**TABLE 4:** Subgroup analyses of berberine on FBG and escape latency.

outcome	subgroup		No. studies	SMD [95% CI]	*I* ^2^	Heterogeneity
FBG	Year	Before 2015	6	−2.48 [−3.16, −1.79]	22.3%	0.266
		After 2015	8	−2.30 [−3.38, −1.22]	80.9%	<0.001
	Animal species	Rats	11	−2.75 [−3.66, −1.86]	76.0%	<0.001
		Mice	3	−1.39 [−2.09, −0.69]	0	0.933
	STZ (mg/kg)	≤40	6	−3.57 [−4.64, −2.49]	57.4%	0.039
		>40	7	−1.70 [−2.43, −0.98]	57.0%	0.030
	Berberine dose (mg/kg)	≤100	8	−2.16 [−3.14, −1.19]	76.8%	<0.001
		>100	6	−2.74 [−3.70, −1.78]	57.9%	0.037
	Duration (week)	≤8	8	−1.94 [−2.77, −1.12]	65.9%	0.004
		>8	6	−3.02 [−4.19, −1.86]	73.2%	0.002
Escape latency	Year	Before 2015	2	−4.32 [−7.06, −1.59]	63.3%	0.099
		After 2015	10	−2.31 [−3.21, −1.41]	78.1%	<0.001
	Animal species	Rats	7	−3.05 [−4.06, −2.03]	66.7%	0.008
		Mice	5	−1.87 [−3.20, −0.55]	79.8%	0.006
	STZ (mg/kg)	≤40	6	−3.04 [−4.33, −1.76]	77.4%	0.001
		>40	4	−3.25 [−5.12, −1.39]	77.1%	0.004
	Berberine dose (mg/kg)	≤100	7	−2.20 [−3.25, −1.14]	76.5%	<0.00
		>100	5	−3.22 [−4.95, −1.50]	82.7%	<0.001
	Duration (week)	≤8	7	−3.25 [−4.65, −1.84]	80.3%	<0.001
		>8	5	−1.94 [−3.12, −0.75]	78.6%	0.001

### 3.6 Sensitivity Analysis

After excluding each trial in turn from the meta-analysis, the results remained consistent with the overall effect size for the effect of berberine supplementation on FBG and escape latency. Although there was no significant effect of any single study on the combined effect size of FBG and escape latency, sensitivity analysis of FBG revealed that the overall effect size was (SMD: −2.15, 95% CI [−2.77, −1.54]) and (SMD: −2.56, 95% CI [−3.19, −1.93]) after omitting ZhouJY et al. and TongMM et al., respectively. For escape latency, omitting any single study had no significant effect on the overall effect size.

### 3.7 Publication Bias

According to the results of Egger’s test, there was a significant publication bias for FBG and escape latency of MWM (*P*Egger < 0.0001). Asymmetry was then corrected using the trim and fill method, with imputing five studies that may have been missed for FBG and escape latency, respectively. The trim and fill analysis indicated that these missed studies did not obviously change the magnitude of the overall pooled effect size. The results from Egger’s test and trim and fill analysis are presented in Table 5.

**TABLE 5:** Results from Egger’s test and trim and fill analysis.

Parameter	Egger’s test *p*-value	Before trim and fill			After trim and fill		
		*p*-value	SMD	No. studies	*p*-value	SMD	No. studies
FBG	< 0.001	< 0.001	−2.41	14	< 0.001	−1.16	19
Escape latency	< 0.001	< 0.001	−2.59	12	0.018	−1.39	17

## 4 Discussion

In this systematic review and meta-analysis, we evaluated the effects and mechanisms of berberine on DCI model animals. A total of 20 studies involving 422 animals were included. According to the results derived from the meta-analysis, berberine could significantly reduce FBG levels and improve learning and memory abilities. The mechanisms underlying the effects of berberine on cognitive function in animals with DCI include regulating glucose and lipid metabolism, improving insulin resistance, anti-oxidative stress, anti-neuroinflammation, inhibiting ER stress, and protecting the cholinergic system. We further conducted a subgroup analysis of the primary outcomes, FBG, and escape latency. The results of the subgroup analysis indicated that animal species and year of publication could be the sources of heterogeneity for FBG. However, because of the small sample sizes, these findings should be prudently interpreted, and more studies are needed to provide more precise evidence. Escape latency, animal species, dose of berberine, duration of treatment, dose of STZ, and year of publication may not be the sources of heterogeneity among studies. Therefore, we speculate that the heterogeneity probably resulted from other differences in the studies, including the design of the research scheme, the standard of successful modeling, characteristics of the sample, and the experimental sample size.

The behavioral test MWM has been widely applied to evaluate the cognitive and behavioral abilities of rodents (Vorhees and Williams, 2006), which usually consists of two parts: the learning trials and the probe trials. Escape latency, defined as the time required to arrive at the platform, is the main content of learning trials. In the probe trials, times of platform crossing and time spent in the target quadrant are used to assess memory ability after removing the platform. Studies have shown that, in the diabetic model animals, the escape latency was significantly prolonged and times of platform crossing and the time spent in the target quadrant were reduced. It was implied that the cognitive function and spatial memory of diabetic model animals were impaired. While berberine supplementation obviously shortened the escape latency, the times of crossing the platform and the time in the target quadrant increased, which indicated that berberine had good neuroprotective potential.

Hyperglycemia is the hallmark of T2DM and the long-term hyperglycemia may have toxicities on neurons through the formation of advanced glycation end products (AGEs) (Lovestone and Smith, 2014). AGEs are products of reactions between proteins or lipids with sugars and accumulate rapidly with aging. Additionally, AGEs can bind to the receptor for advanced glycation end products (RAGE) to produce reactive oxygen species (ROS), which could cause oxidative stress in neurons, activate the p38 mitogen-activated protein kinase (MAPK) pathway, upregulate the NF-κB expression, and lead to an inflammatory response (Adhikary et al., 2004). Studies have demonstrated that berberine can significantly reduce FBG, 2hBG of OGTT, and 2hBG of ITT, thereby reducing the production of AGEs. In addition, berberine can also downregulate the expression of RAGE and p38 MAPK and reduce oxidative stress and inflammatory damage. Insulin resistance (IR) is an important pathogenesis of T2DM. In the central nervous system (CNS), insulin, insulin-like growth factor 1 (IGF-1), and their associated receptors are highly concentrated within the hippocampus, hypothalamus, and cortex, and their combination leads to IR substrate protein (IRS-1) phosphorylation (Bedse et al., 2015). However, chronic peripheral hyperinsulinemia downregulates blood–brain barrier insulin receptors and decreases the amount of insulin transported into the CNS (Kellar and Craft, 2020). Then, the IRS-activated phosphoinositide 3-kinase (PI3K)/protein kinase B (Akt) pathway is inhibited, and the activity of glycogen synthase kinase (GSK-3β) in downstream sites of the pathway is elevated, thus affecting the transport of glucose transporter 3 (GLUT3) into the brain, which impairs amyloid clearance and results in cognitive decline (Zaulkffali et al., 2019; Leao et al., 2020). Current studies suggested that the serum insulin index of experimental diabetic animals decreased significantly, and the phosphorylation levels of PI3K/Akt/GSK-3β were reversed after berberine supplementation. Berberine could promote glucose uptake and metabolism in the brain by upregulating the expression of GLUT3. However, only one study reported berberine elevated central insulin levels compared with diabetic model animals (Wang et al., 2019). Therefore, it may be difficult to determine the effects of berberine on central insulin levels.

Lipid metabolism plays an important role in maintaining the structure and function of brain neurons. DM patients are usually accompanied by lipid dysmetabolism, which can induce the occurrence of neurodegenerative diseases, such as AD (Chornenkyy et al., 2019). The brain is one of the most lipid-rich organs, and the increase in total cholesterol (TC) is a significant risk factor for cognitive impairment (Anstey et al., 2017; Chornenkyy et al., 2019). Studies demonstrated that high levels of cholesterol modulate Aβ levels by affecting the activities of beta-secretase 1 (BACE1) (Liu et al., 2007; Beel et al., 2010). Experimental data suggested that berberine can ameliorate lipid metabolism by reducing the levels of TC, triglyceride (TG), and low-density lipoprotein cholesterol (LDL-C) and accelerate the clearance of APP, Aβ, and BACE1 in the brain.

Oxidative stress is closely related to the pathogenesis of DCI. When the body is exposed to harmful stimulation, ROS are overproduced. It causes lipid peroxidation of nerve cells and impairs the ability of antioxidant damage. Multiple studies showed that the levels of ROS and MDA increased significantly while the levels of GSH and SOD decreased significantly in the hippocampi of diabetic animals (Ma et al., 2012b; Moghaddam et al., 2014; Adefegha et al., 2021; Tian et al., 2021). Meanwhile, nuclear factor erythroid-2 related factor 2 (Nrf2) plays a pivotal defensive role against oxidative stress *via* eliciting the transcription of anti-oxidative genes encoding heme oxygenase-1 (HO-1) and NADPH quinine oxidoreductase 1 (NQO1) (Ghanim et al., 2021). This study found that berberine can reduce oxidative stress by activating the Nrf2/HO-1/NQO1 pathway. Moreover, the inflammatory response is present throughout the development of diabetes and its complications. It is generally thought that inflammation causes neurodegeneration because microglia and astrocytes in the hippocampus are activated and release large amounts of inflammatory factors (Kaur et al., 2019). In the physiological state, microglia and astrocytes are inactive and only release a small amount of cytokines. Once stimulated by hyperglycemia, neuroglia are activated rapidly and release many pro-inflammatory and cytotoxic factors, leading to the activation of MAPK and AGEs/RAGE/NF-κB signal pathways (Tobon-Velasco et al., 2014; Liu Y. et al., 2018). Besides, chronic inflammation will change the permeability of the blood–brain barrier, resulting in Aβ deposition and tau protein phosphorylation, which further exacerbates inflammatory response and synaptic damage (Michalicova et al., 2020; McLarnon, 2021). Berberine can regulate the expression of AGEs, RAGE, and NF-κB; reduce the release of pro-inflammatory cytokines such as TNF-α, IL-1β, and IL-6; and attenuate Aβ deposition and p-tau protein expression to delay cognitive impairment. Moreover, the occurrence and development of various inflammatory responses are closely correlated to endoplasmic reticulum (ER) stress, which is considered the pathogenic factor of many chronic neurodegenerative diseases (Zhang et al., 2015; Liu et al., 2020). It is reported that high glucose increases ER stress levels, and activation of ER stress pathway could participate in diabetes-induced neuronal apoptosis and cognitive decline (Kong et al., 2018; Escribano-Lopez et al., 2019). The specific mechanism is as follows: ER stress induces the dissociation of protein kinase R-like ER kinase (PERK), inositol-requiring enzyme 1α (IRE1α), and glucose-regulated protein 78 (GRP78), further activating the c-Jun N-terminal kinase (JNK) and PERK signaling pathway. In addition, PERK phosphorylates its substrate eukaryotic translation initiation factor 2α (eIF2α), then activating pro-apoptotic factor C/EBP homologous protein (CHOP), which eventually leads to neuronal apoptosis (Ettcheto et al., 2019). Our findings showed that berberine can decrease the expression of IRE1α, CHOP, and p-PERK by affecting the signal transduction of PERK and regulate the neuronal apoptosis by inhibiting the expression of ER stress to improve the ability of learning and memory of diabetic animals.

In addition to the above, the disorder of the central cholinergic nervous system is one of the important causes of DCI. The cholinergic system of CNS is involved in memory, and acetylcholine (ACh) is the main neurotransmitter responsible for learning and memory in the CNS. The expression of acetylcholinesterase (AChE) is partly regulated by insulin. Under the condition of long-term IR, the activity of AChE in the hippocampus is significantly enhanced (Hamed, 2017; Kim et al., 2017). Our data suggested that berberine can prevent the damage to the cholinergic system by decreasing the level of AChE, thereby improving the ability to learn and memory.

In the current review, we included all of the conducted meta-analyses focusing on the effects of berberine on blood glucose, cognitive function, and relevant mechanisms. However, there are some limitations of this study that should be noted. First, in terms of the quality of studies, none of the studies have described the method used to conceal the allocation sequence, blind intervention, and random outcome assessment. Some studies did not provide detailed baseline characteristics. In addition, given that the methodological quality of some studies was not sufficient to make definitive judgments about the risk of bias, the results of this study should be interpreted with caution, and more high-quality studies will be needed in the future. Second, the high degree of heterogeneity in the combined results reduces the reliability of the results (Prady et al., 2014). Although we attempted to explore potential sources of heterogeneity using our prespecified subgroup analysis, this did not appear to work well. The different study protocols and intervention processes in animal experiments may be key potential factors contributing to high heterogeneity. Therefore, we suggest that, in future studies, researchers should pay more attention to the rigor of research design and provide adequate information for experimental research. Third, because available data for some indicators existed only in individual studies, we did not conduct further meta-analysis on the relevant indicators. These indicators will require more attention in the future. Obviously, much work still needs to be done to adequately address the shortcomings of the available literature.

## 5 Conclusion

The present meta-analysis revealed that berberine could lower blood glucose levels and improve learning and memory abilities. The underlying mechanisms of these protective effects include regulating glucose and lipid metabolism, improving insulin resistance, anti-oxidation, anti-neuroinflammation, inhibiting ER stress, and protecting the cholinergic system. However, the validity of the research results may be undermined by the low methodological quality and publication bias in the included studies. More rigorous experiment designs and more comprehensive studies are required to test the protection of berberine against DCI in the future.

## Data Availability

The original contributions presented in the study are included in the article/supplementary material. Further inquiries can be directed to the corresponding author.
